# First detection of *Dickeya fangzhongdai* in Italy: double-edged role from virulent on vegetables to potent VOC-mediated anti-*Xylella fastidiosa* activity

**DOI:** 10.3389/fmicb.2025.1655235

**Published:** 2025-08-05

**Authors:** Miloud Sabri, Kaoutar El Handi, Khaoula Mektoubi, Antonio Trani, Orges Cara, Toufic Elbeaino

**Affiliations:** ^1^International Centre for Advanced Mediterranean Agronomic Studies (CIHEAM of Bari), Bari, Italy; ^2^Department of Soil, Plant and Food Sciences, University of Bari Aldo Moro, Bari, Italy; ^3^National Research Council of Italy (CNR), Institute for Sustainable Plant Protection (IPSP), Naples, Italy

**Keywords:** bacterial soft rot, antagonistic activity, Brassica, whole-genome sequencing, SPME/GC–MS

## Abstract

In this study, we report the first detection of *Dickeya fangzhongdai* (*D. fangzhongdai*) in Italy, which was isolated from an asymptomatic cauliflower plant in Valenzano (Apulia region), as part of an effort to isolate antagonistic bacteria against *Xylella fastidiosa* subsp. *pauca* (*Xf*p). Results of the preliminary antagonistic screening showed that one isolate (C7) has a strong activity against *Xf* on BCYE agar plates. The Illumina whole-genome sequencing and bioinformatic analysis showed that C7 genome consists of a single circular chromosome of 5,045,655 bp in size, with an average nucleotide identity (ANI) exceeding 98% when compared with other *D. fangzhongdai* strains reported in the GenBank; thereby confirming its taxonomic allocation within the *D. fangzhongdai* species. Furthermore, multi-locus sequence analysis employing concatenated sequences of the *atpD*, *gyrB*, *infB*, and *rpoB* genes showed that the strain C7 is phylogenetically close to *D. fangzhongdai* strain B16 from Slovenia (acc. no: NZ_CP087226). The strong antagonistic activity of C7 against *Xf* was also observed under physically separated conditions using a cut agar barrier, suggesting the involvement of volatile antimicrobial compounds (VOCs). Thus, headspace solid-phase microextraction and gas chromatography coupled with mass spectrometry (SPME/GC–MS) analysis revealed that the strain C7 emits 17 distinct VOCs during incubation in YPG broth, suggesting that strain C7 employs VOC-mediated inhibition as a defense mechanism to suppress bacterial competitors. Pathogenicity assessment showed that strain C7 induces severe soft rot in potato tubers, onion bulbs, and pear fruits, as well as, for the first time, causes browning of vascular tissues in cauliflower plants and blackleg in Havana tobacco plants. The prior detection of *D. fangzhongdai* in the Netherlands and its present identification in Italy on new hosts (cauliflower and tobacco) raise significant concerns about its further establishment, expanded host range, and phytosanitary implications for European agriculture.

## 1 Introduction

*Dickeya* is a genus of Gram-negative, facultatively anaerobic, rod-shaped bacteria that includes several pathogenic species responsible for devastating diseases in a wide range of economically important crops, vegetables, and ornamental plants worldwide (Kmoch et al., 2024). As a member of the Soft Rot Pectobacteriaceae (SRP), *Dickeya* belongs to a group of bacteria characterized by their ability to produce a variety of plant cell wall-degrading enzymes (PCWDEs), leading to cell lysis and the release of cellular contents that serve as nutrients for their propagation and population growth (Zhou et al., 2024; Babińska-Wensierska et al., 2025). Diseases caused by *Dickeya* species include soft rot, blackleg in potato, foot rot in rice, and bleeding canker in pear (Charkowski, 2018). The severity of these diseases varies depending on environmental factors, pathogen inoculum, and plant susceptibility, causing crop losses up to 40% (Elhalag et al., 2024). *Dickeya* spp., likely *Dickeya dianthicola* (*D. dianthicola*), were first reported on potato in Netherlands during 1970s and have since been detected in many other European countries, where they have severely impacted the potato industry (Toth et al., 2011; Ge et al., 2021a).

Over the past two decades, *Dickeya*-associated diseases have become increasingly widespread, with newly identified species and expanding host ranges posing a significant threat to global agricultural production (Alič et al., 2024; Zhou et al., 2024). For example, the emergence of *D. solani* has caused persistent blackleg outbreaks in Finland for over a decade, resulting in substantial yield losses and the downgrading of seed potato lots (Degefu et al., 2013). Similarly, an outbreak of blackleg and soft rot of potato, initiated in Maine in 2015 and primarily associated with *D. dianthicola*, has since caused significant economic losses across the northeastern and mid-Atlantic United States (Jiang et al., 2016; Ge et al., 2021b). Degefu (2024) projected that losses from blackleg and soft rot will escalate under changing climatic conditions, primarily due to the emergence of new, highly aggressive and difficult-to-control species of *Dickeya*, which are becoming increasingly frequent and widespread.

A recently emerged *Dickeya* species is *D. fangzhongdai*, which has caused severe diseases in recent years on various ornamental and fruit tree species, most notably pear bleeding canker in China and orchid soft rot in both Asia and Europe (Tian et al., 2016; Zhou et al., 2024). *D. fangzhongdai* was originally identified in China in 2016 as the causal agent of bleeding canker in Asian pear (*Pyrus pyrifolia*) and is the first reported *Dickeya* species to infect trees, where the symptoms do not appear as a soft rot, but rather as bleeding canker necrosis, suggesting a broad ecological adaptability and an expanding host range (Tian et al., 2016). Since then, it has been associated with soft rot of onion in the USA (Ma et al., 2020), orchids in Canada (Zhou et al., 2021), taro (*Colocasia esculenta*) in China (Huang et al., 2021), and banana in both China and Ecuador (Yang et al., 2022; Toaza et al., 2025). However, data on the economic impact, distribution, and full host range of *D. fangzhongdai* remain limited, likely due to its recent emergence, restricted geographic surveillance, and diagnostic challenges arising from its similarities with other *Dickeya* species. In this study, and to the best of our knowledge, this is the first confirmed report of *D. fangzhongdai* in Italy, where it was isolated from cauliflower stalks and identified as the causal agent of soft rot in pear, onion, and potato, as well as browning of vascular tissues in cauliflower plants and blackleg in Havana tobacco plants. The detection of this species in a new geographic area and affecting new hosts raises significant concerns regarding its potential establishment, host range, and phytosanitary implications for Italian agriculture. Furthermore, it was found to emit different volatile organic compounds (VOCs) with *in vitro* anti-*Xylella fastidiosa* subsp. *pauca (Xfp)* activity. *Xfp* is a xylem-limited plant pathogenic bacterium responsible for destructive diseases such as olive quick decline syndrome (OQDS), leading to significant and irreversible cultural damages (Saponari et al., 2019). In Italy, OQDS affected nearly 54,000 hectares of olive orchards between 2013 and 2019 (Frem et al., 2021), resulting in an economic loss of nearly €390 million (Scala et al., 2020). The management of *Xfp* remains highly challenging due to its wide host range, systemic colonization, and lack of effective chemical controls, highlighting the urgent need for alternative and sustainable solutions (Beretta et al., 2022). In this context, the present study reports the double-edged role of *D. fangzhongdai*, from being virulent to vegetables to exhibiting potent VOC-mediated antagonism against *Xfp*, and highlights for the first time the potential of VOCs as a promising novel strategy for the control of *Xfp*.

## 2 Materials and methods

### 2.1 Collection of plant samples and bacterial isolation

In October 2024, cauliflower (*Brassica oleracea* var. *botrytis*) and broccoli (*Brassica oleracea var. italica*) samples were collected from a weekly open-air market located at 41.04378° N, 16.88420° E in Valenzano (BA, Italy) for the purpose of isolating antagonistic bacteria against *Xfp* and *Xanthomonas campestris* pv. *campestris* (*Xcc*). The choice of cauliflower and broccoli, both susceptible to *Xcc*, which is phylogenetically related to *Xf*, was intended to enhance the likelihood of isolating antagonistic bacteria with potential activity against *Xf*. Stalk tissues of three plants from each broccoli and cauliflower species were used for bacterial isolation. Briefly, 1 g of plant tissue was macerated in 4 mL of sterile distilled water, followed by six 10-fold serial dilutions. Aliquots of 50 μL from each dilution were plated onto yeast extract peptone glucose agar (YPGA) (5.0 g/L yeast extract, 5.0 g/L peptone, 10.0 g/L glucose, and 15.0 g/L agar) for bacterial isolation. Plates were incubated at 28°C for 24 h, after which morphologically distinct colonies were purified and stored at −80°C in 25% glycerol prepared in YPG broth for further study.

### 2.2 Evaluation of antagonistic activity, DNA extraction, and whole-genome sequencing

Bacterial isolates obtained from cauliflower and broccoli plant samples were initially subjected to antagonistic screening against *Xcc* strain CFBP 1710 and *Xfp* strain A0PT1 (Sequence Type, ST53) using dual-plate confrontation assays, following the methodology described by Sabri et al. (2025). One isolate, subsequently identified as *D. fangzhongdai* strain C7, was prioritized for whole-genome sequencing (WGS) due to its strong antagonistic activity against *Xf* on BCYE agar plates (Wells et al., 1981), to determine its taxonomic identity. The antagonistic effect of strain C7 against *Xfp* was assessed as follows: four 30 μL drops of *Xfp* suspension (OD₆₀₀ = 0.32), prepared in sterile water, were placed along the upper section of BCYE agar plates, with approximately 1.5 cm spacing between drops. These drops were allowed to slowly descend to the opposite side, forming four parallel rows of *Xfp* cultures. After the drops had dried under a laminar flow hood, 10 μL of C7 suspension (OD₆₀₀ = 0.2), prepared in sterile water from an overnight culture, was deposited at the center of one *Xfp* row. Sterile water served as negative control and the plates were incubated for 10 days at 28°C. Genomic DNA of strain C7 was extracted using the cetyltrimethylammonium bromide (CTAB) method, following the protocol described by Wilson (2001). The WGS was performed by Eurofins Genomics (Ebersberg, Germany), using Illumina HiSeq 2,500 paired-end sequencing technology, with an average read length of 150 bp. The reads were quality checked and trimmed, and *de novo* assembled using the Geneious Prime 2025.1.1 software (Biomatters, Ltd., San Diego, CA, USA). The complete genome of strain C7 was annotated using Prokka v1.14.6^1^. Clustered Regularly Interspaced Palindromic Short Repeats (CRISPRs) were identified using CRISPRCas++^2^, and prophage genes were predicted using the online tool PHASTER^3^. Additionally, genomic islands (GIs) were predicted using IslandPath-DIMOB, as implemented in IslandViewer 4.^4^

### 2.3 Multilocus sequence analysis (MLSA)

To determine the phylogenetic position of strain C7, MLSA was performed using four housekeeping genes (*atpD*, *gyrB*, *infB*, and *rpoB*) as described by Chen et al. (2024). Briefly, DNA sequences of the four housekeeping genes were aligned using the Geneious Alignment algorithm within the Geneious software platform. Concatenated nucleotide sequences of the four genes were used to construct a phylogenetic tree using the maximum likelihood method derived from MEGA 11 software, with 1,000 bootstrap replicates to assess branch support. Sequences from representative *Dickeya* species were included for comparison.

### 2.4 VOC production by *Dickeya fangzhongdai* C7 for bacterial antagonism

The strong antagonistic activity of C7 against *Xfp*, observed under both direct contact and physically separated conditions using a cut agar barrier, suggested the involvement of volatile antimicrobial compounds. Therefore, the production of VOCs by C7 was investigated using headspace solid-phase microextraction and gas chromatography coupled with mass spectrometry (SPME/GC–MS). Briefly, 5 mL of YPG broth were placed in SPME glass vials of 20 mL and inoculated with 200 μL of C7 suspension (10^8^ CFU/mL). The vials were incubated at 28°C for 24, 48, and 72 h. At each time point, three replicates per incubation period, along with a control containing only YPG broth, were placed on a robotic autosampler Pal Combe XT. The SPME extraction was performed at 50°C constant temperature, 2 min of equilibration and agitation, 15 min of extraction by exposing a SPME fiber DVB/CAR/PDMS, 50 μm (DVB layer), 30 μm (CAR/PDMS layer) (Supelco) in the headspace. The VOCs were then desorbed in a split injector of a GC/MS system made of a Clarus 680 GC equipped with an VF-WAXms fused silica capillary column (20 m x 0.1 mm and 0.1 μm film thickness) and interfaced with a single quadrupole mass spectrometer Clarus SQ8C (Perkin Elmer). Mass spectra of target compounds were obtained by electron impact ionization with standardized ionization energy of 70 eV. Helium 5.5 was used as a carrier gas at a constant flow rate of 0.37 mL/min. The injection was performed in splitless (closed split valve for 0.1 min) at 230°C. The oven temperature was programmed from 60°C to 240°C at 20°C/min, then hold to 240°C for 2 min. Transfer line and source temperatures were set at 250°C. Data were collected in full scan mode in the range 33–300 m/z. Qualitative results include compound identification and area percentage of related peak in the total ions chromatogram. Compound identification was performed by mass spectra (MS) search in NIST and Wiley databases. The retention index for the identified compounds was calculated according to van den Dool and Kratz (1963) and, to the best of our knowledge, for the first time using the innovative cross linked, polar stationary phase of the chromatographic column, with fast chromatography. Thus, the reported information can be useful in the future research for identification purposes.

### 2.5 Pathogenicity tests

The pathogenicity of *D. fangzhongdai* strain C7 was tested on cauliflower, broccoli, and Havana tobacco (*Nicotiana tabacum*) plants, as well as on potato tubers (*Solanum tuberosum* cv. Monalisa), pear fruits (*Pyrus communis L.* cv. Comice), and onion bulbs (*Allium cepa* cv. Belendina). For cauliflower, broccoli, and Havana tobacco plants, 50 μL of C7 suspension (10^8^ CFU/mL) were injected into the stems using a sterile syringe. Ten one-month-old plants from each species were inoculated, and an additional 10 plants injected with sterile water served as negative controls. Plants were maintained in a controlled quarantine laboratory environment at 26°C for 22 days. In addition, 10 potato tubers, 10 pear fruits, and 10 onion bulbs were surface disinfected by immersion in 70% ethanol for 3 min, followed by rinsing with sterile distilled water. Wounds approximately 2 mm in diameter were created using a sterile scalpel, and 50 μL of C7 suspension (10^8^ CFU/mL) was inoculated into each wound. The inoculated and control samples were subsequently placed in sterile, high-humidity chambers and incubated at 28°C for 5 days. All experiments were conducted in duplicate.

### 2.6 Persistence of *Dickeya fangzhongdai* strain C7 in cauliflower and broccoli plants

To evaluate the persistence of C7 within cauliflower and broccoli plants 22 days post-inoculation (dpi), stems of three plants from each species were separately ground in 2 mL of sterile water and centrifuged at 1,000 g for 5 min to remove plant debris. The resultant supernatant was diluted six times using a 10-fold serial dilution, and 50 μL of each dilution was plated onto YPGA plates. The plates were incubated at 28°C for 24 h and C7-like colonies were purified on YPGA plates and subjected to PCR testing using *D. fangzhongdai*-specific primers (DF-F:5′-GTTGGCCTTCGAACTCGGTA-3′; DF-R:5′-TCAAACTGCGCCGGATAACT-3′), which were designed in this study using Geneious software. The PCR cycles consisted of an initial denaturation step at 94°C for 5 min; 35 cycles of 94°C for 30 s, 60°C for 30 s, and 72°C for 30 s; and a final elongation step at 72°C for 5 min.

### 2.7 Statistical data analysis

The data related to the VOCs determination obtained by SPME-GC/MS were analyzed using SPSS v22 (IBM). The mean values of the area percentage related to each identified compound together with the total peaks area amount at different incubation times were compared using one-way ANOVA followed by Tukey’s *post hoc* test, with a significance threshold of *p* < 0.05.

## 3 Results

### 3.1 Complete genome sequence of *Dickeya fangzhongdai* strain C7

**Figure 1:**
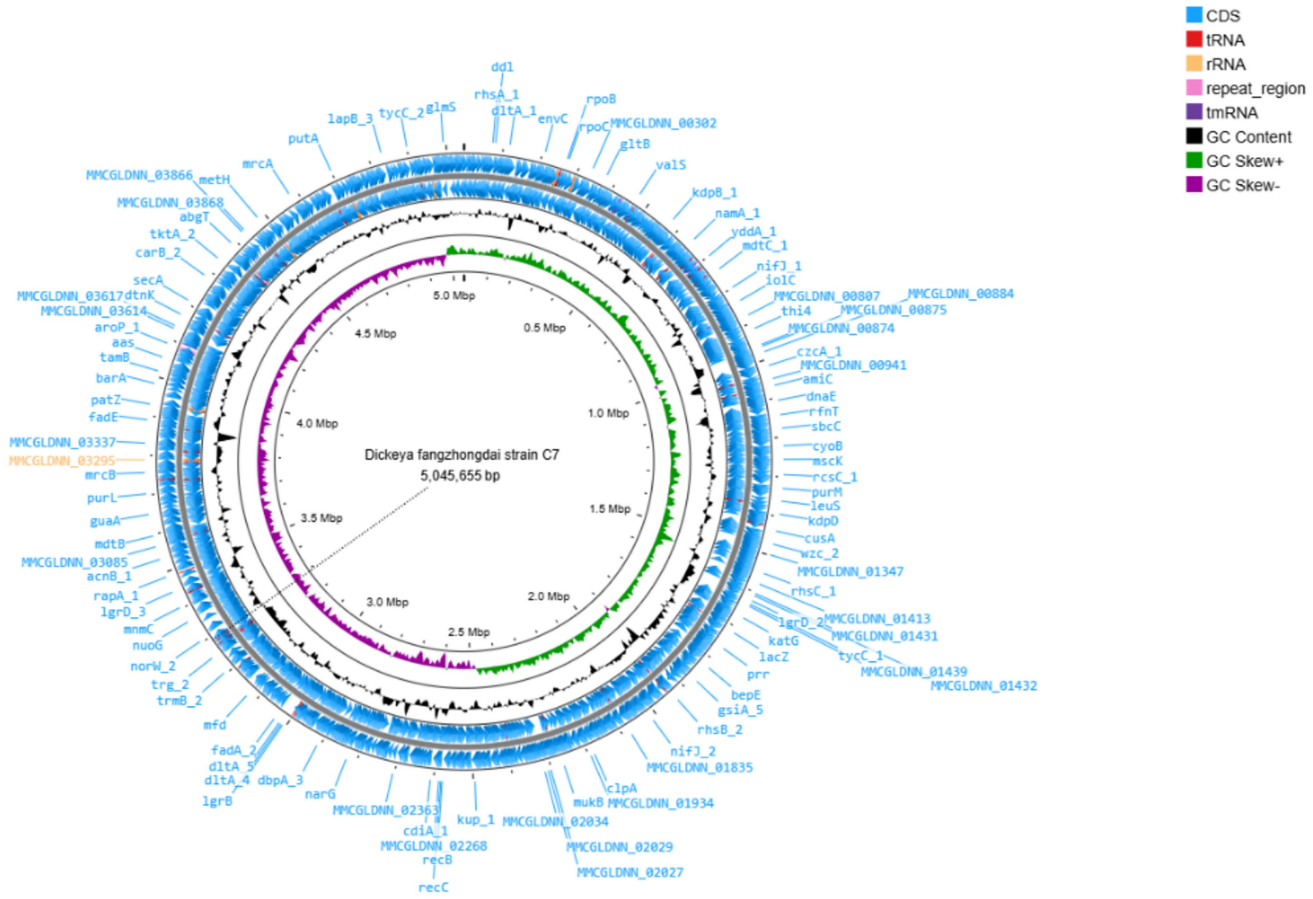
Circular visualization of the complete genome of *Dickeya fangzhongdai* strain C7, generated using Proksee (https://proksee.ca/). The map displays predicted coding sequences (CDSs), functionally annotated proteins, tRNA and rRNA genes, as well as GC content and GC skew.

**Figure 2:**
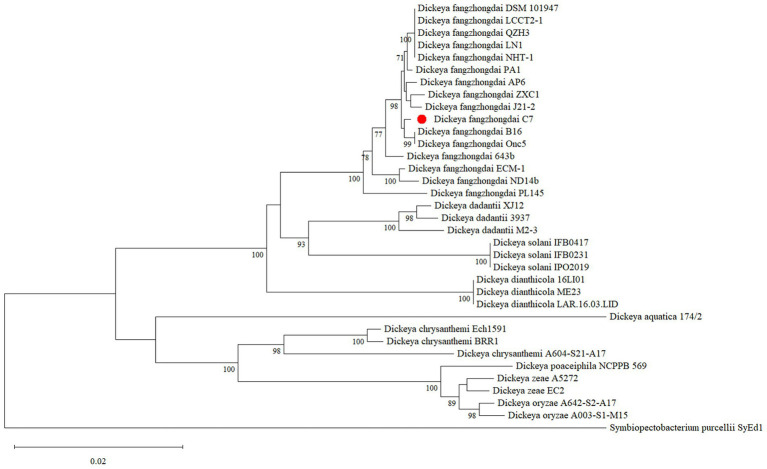
Maximum-likelihood phylogenetic tree illustrating the phylogenetic position of *Dickeya fangzhongdai* strain C7 among closely related *Dickeya* species. The phylogenetic tree was constructed based on the alignment of concatenated sequences from the four housekeeping genes (*atpD*, gyrB, *infB*, and *rpoB*) using MEGA 11 software. Bootstrap values (based on 1,000 replications) of >70% are displayed at the branch points. The scale bar represents 0.020 substitutions per nucleotide position.

The complete genome of *D. fangzhongdai* strain C7 was sequenced using Illumina HiSeq technology, yielding 259,846 high-quality reads, corresponding to approximately 29,844 kb of raw sequence data. *De novo* assembly was performed using Geneious assembler, and the generated contigs were mapped against eight related reference genomes of *D. fangzhongdai* (accession no: CP020872.1, CP080400.1, CP087226.1, CP092458.1, CP092460.1, CP094338.1, CP107067.1, and CP109768.1). The assembled genome revealed a single circular chromosome with a total length of 5,045,655 bp and a GC content of 56.9%. The average nucleotide identity (ANI) value between C7 and other *D. fangzhongdai* strains was over 98.0%, exceeding the 95% species demarcation threshold, and thereby confirming its taxonomic placement within the species. The annotation of the genome predicted 4,311 coding sequences (CDS), 25 rRNA genes, 81 tRNA genes, and one tmRNA gene (Figure 1). In addition, the genome of C7 harbors 9 CRISPR arrays, two distinct CRISPR-associated (Cas) gene clusters, 10 GIs, and 12 prophage gene clusters. The complete genome sequence of C7 has been deposited in the GenBank under the acc. no: CP193488. Furthermore, the phylogenetic analysis placed strain C7 close to strain B16 from Slovenia (acc. no: NZ_CP087226), indicating a high level of genetic similarity and suggesting a close evolutionary relationship between the two strains (Figure 2).

### 3.2 Morphological characteristics and anti-Xylella potential of *Dickeya fangzhongdai* strain C7

**Figure 3:**
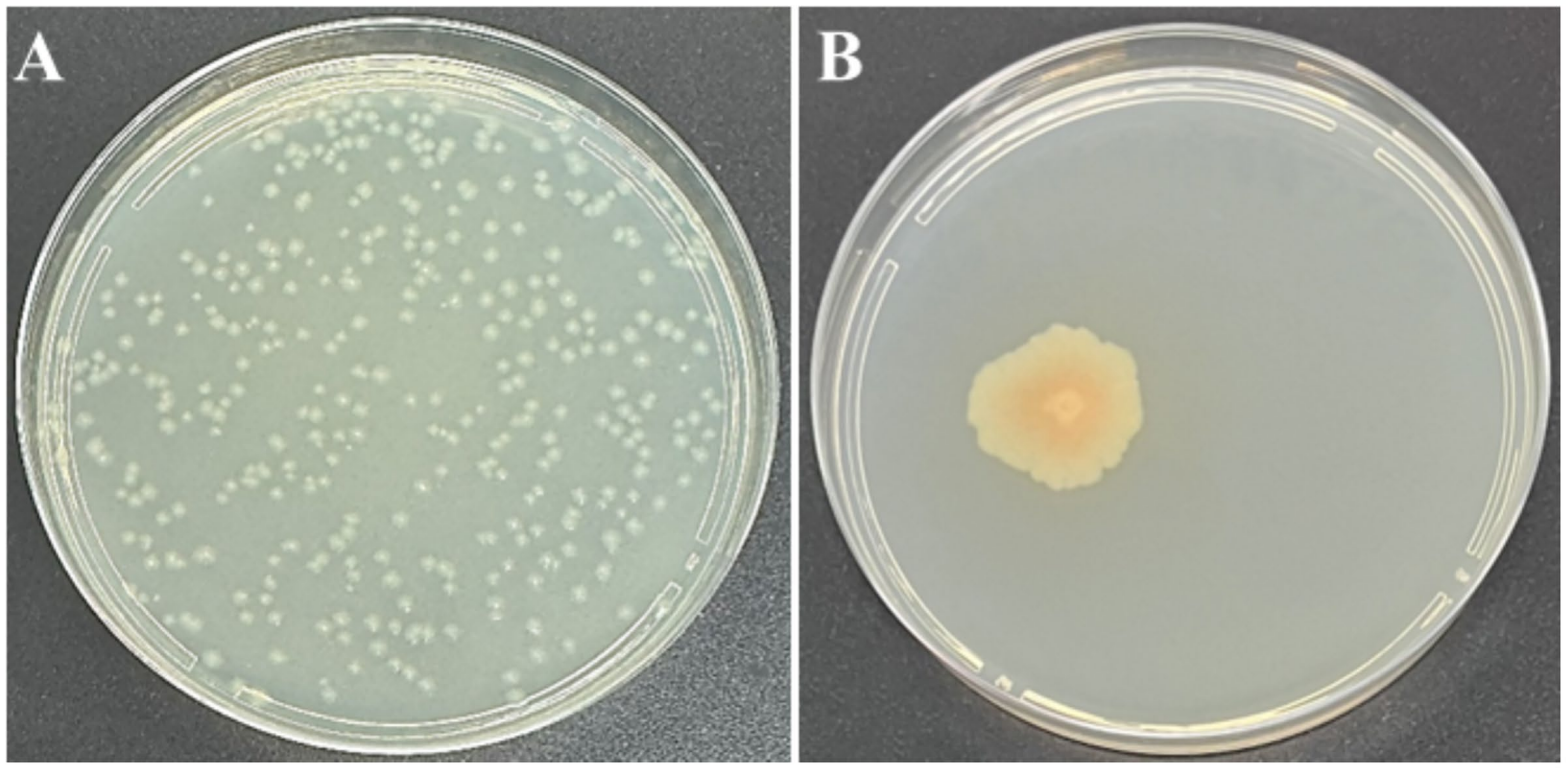
Morphological characteristics of *Dickeya fangzhongdai* strain C7 colonies on YPGA medium after 24 h **(A)** and 72 h **(B)**. In both panels, plates were inoculated with 100 μL of bacterial suspension containing ~10^6^ CFU/mL. Panel **A** (from a 10^−3^ dilution) shows numerous small, well-separated colonies after 24 h of incubation, while Panel **B** (from a 10^−6^ dilution) displays a single, large colony developed after 72 h due to the spatial isolation of a single CFU.

**Figure 4:**
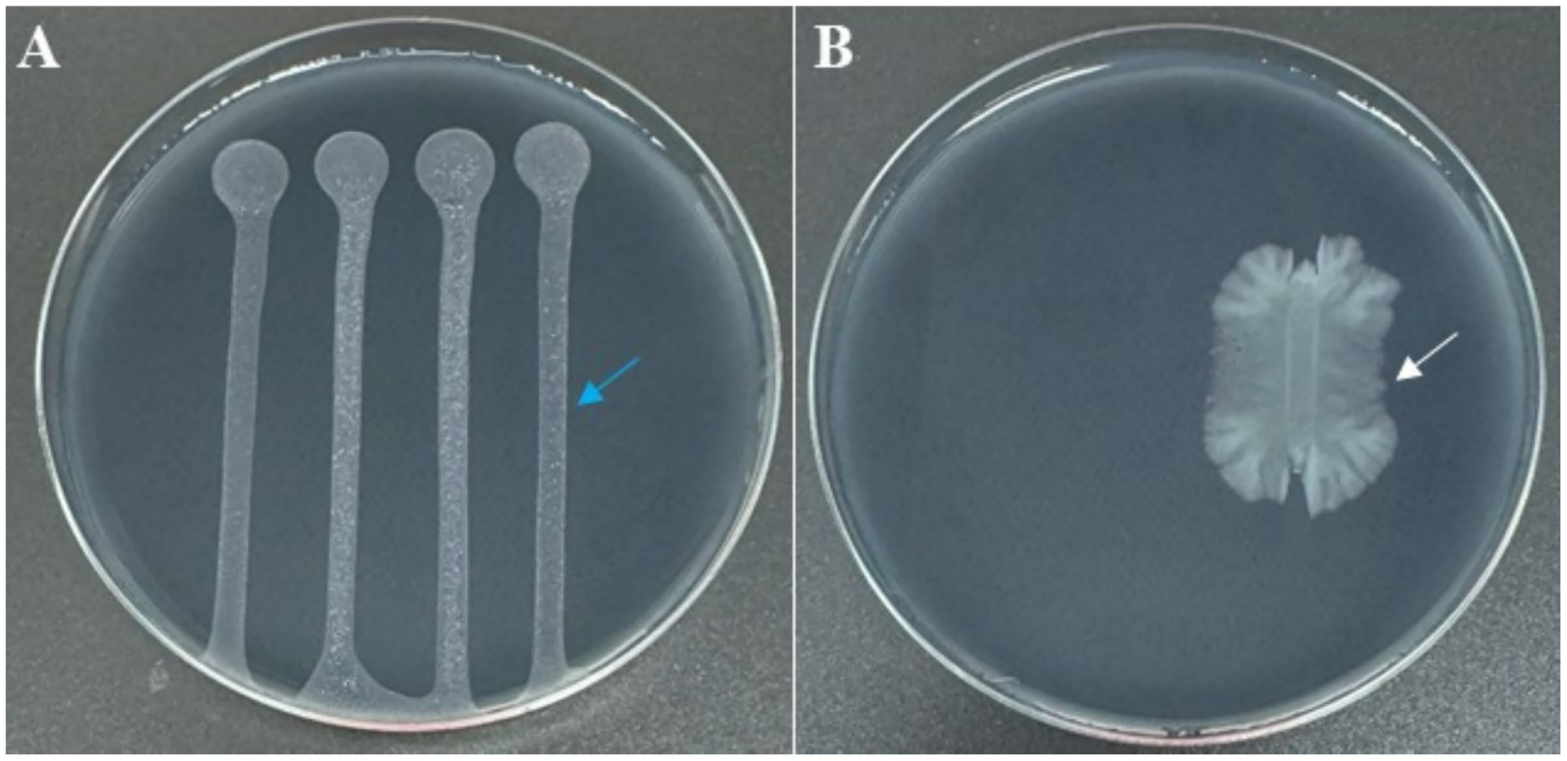
BYCE plates showing the antagonistic activity of *Dickeya fangzhongdai* strain C7 against *Xylella fastidiosa* subsp. *pauca*. **(A)** *Xf* treated with sterile water (blue arrow), showing normal growth with no inhibition. **(B)** *Xf* challenged with C7 (white arrow), resulting in complete inhibition of *Xf*.

Colony morphology of strain C7, grown on YPGA plates for 24 h, was characterized by a circular shape with a smooth glistening surface, cream to off-white in color, and a convex elevation. Colonies reached 2–4 mm in diameter with entire to slightly undulate margins (Figure 3A). After 72 h of incubation, pigmentation was observed, with colonies showing a pale yellow to cream coloration and a distinct bright orange central zone developed. The average colony diameter ranged from approximately 1 to 2.5 cm (Figure 3B). Remarkably, strain *C7* showed a strong antagonistic effect against *Xfp* under *in vitro* conditions, in contrast to the uninhibited growth observed in non-challenged *Xfp* cells (Figure 4A). This complete inhibition suggested the potential synthesis of antibacterial metabolites by strain C7.

### 3.3 VOCs produced by *Dickeya fangzhongdai* C7

**Figure 5:**
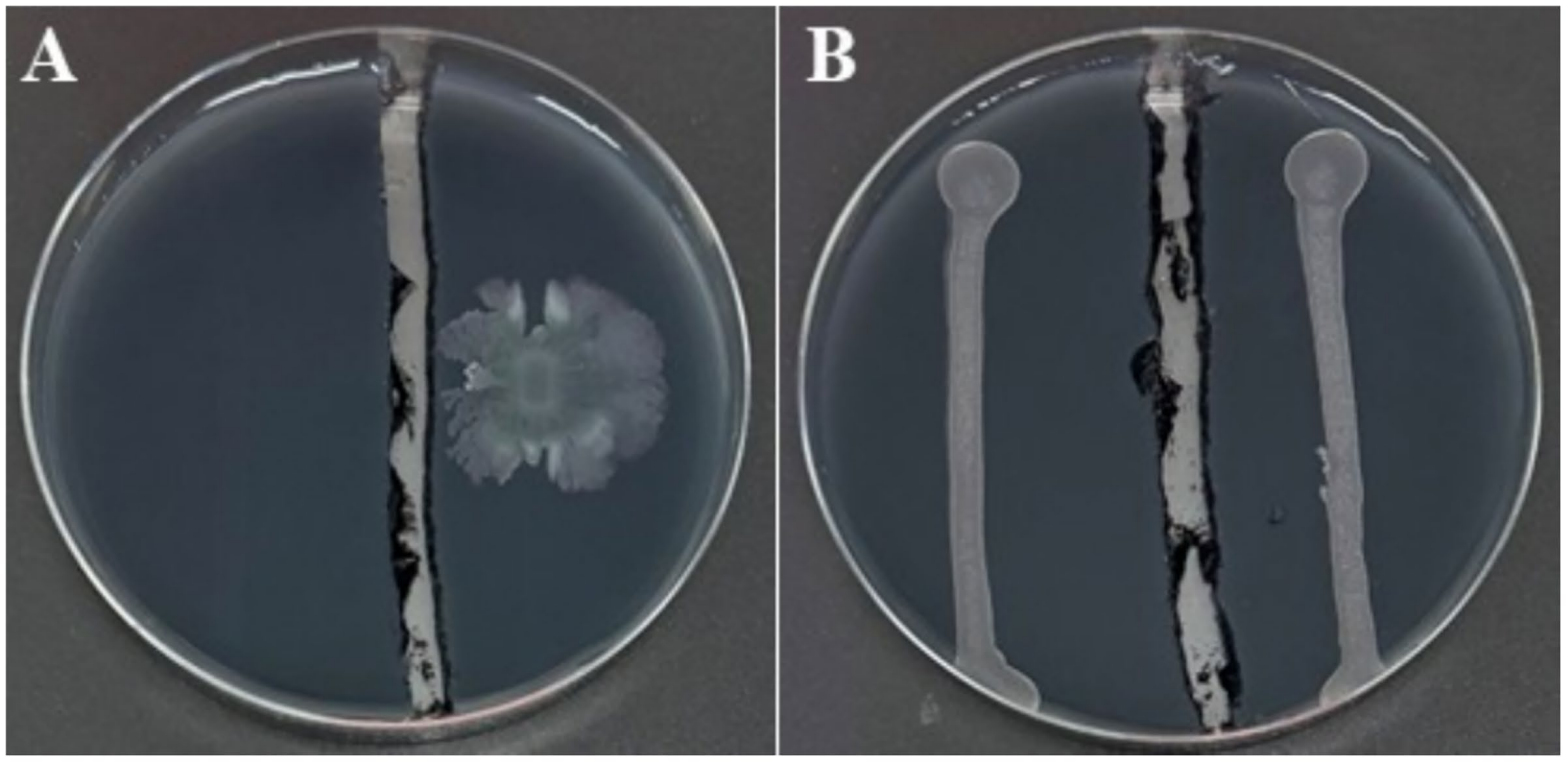
BYCE plates showing the antibacterial activity of *Dickeya fangzhongdai* strain C7 against *Xylella fastidiosa* subsp. *pauca* under physically separated conditions using a cut agar barrier. **(A)** *Xf* challenged with C7. **(B)** *Xf* treated with sterile water.

**Figure 6:**
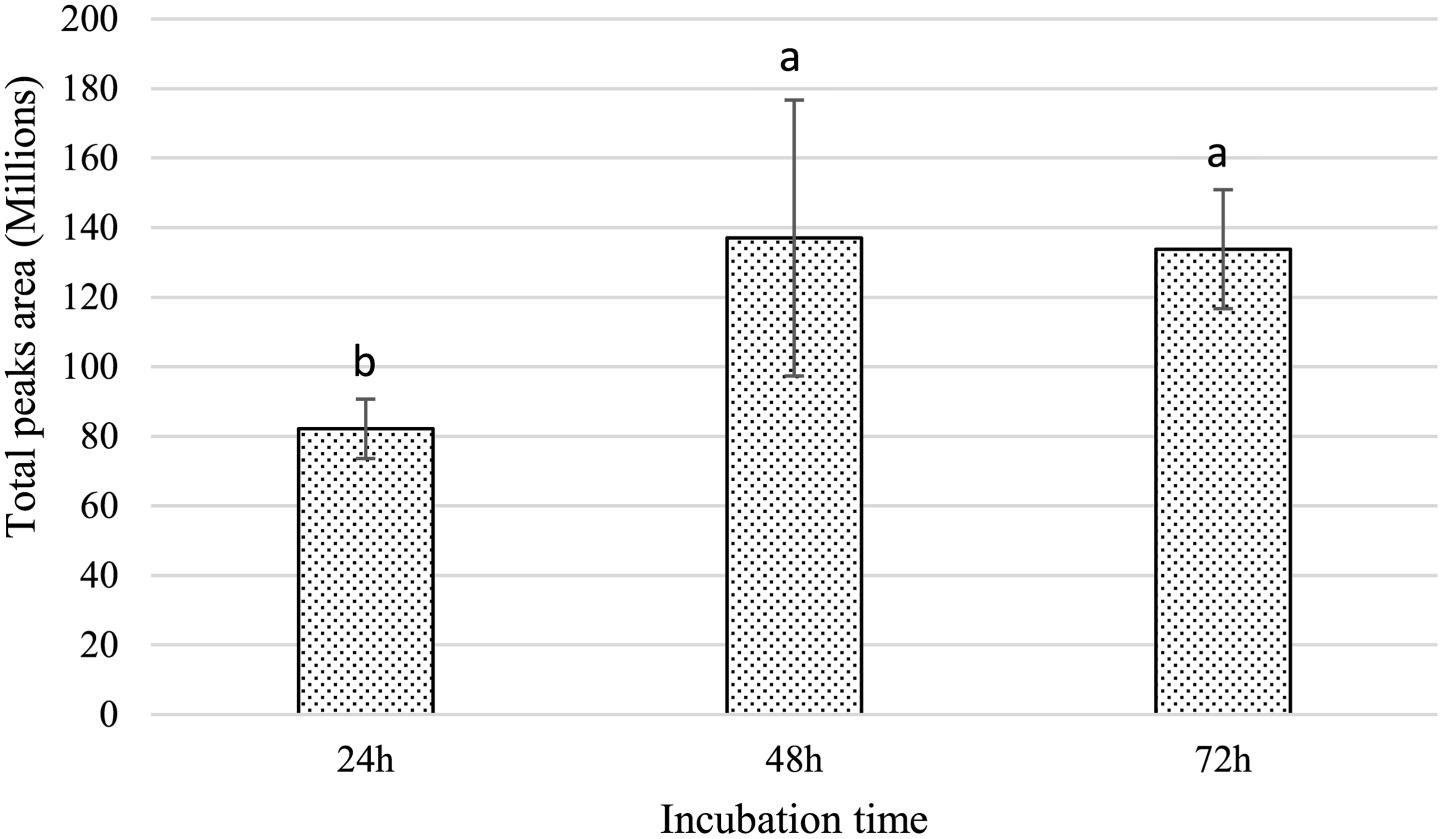
Histogram shows the increase in the cumulative peak area of VOCs detected in the total ion chromatograms. Error bars represent the standard deviation (SD). Different letters above each bar indicate statistically significant differences according to one-way ANOVA followed by Tukey’s *post hoc* test (*p* < 0.01).

The dual-culture assay was performed on solid medium, wherein the agar separating the two bacterial strains was physically removed, thereby disrupting potential diffusion pathways for non-volatile metabolites through the agar matrix. Notwithstanding this spatial segregation, the growth of *Xfp* remained entirely inhibited (Figure 5), suggesting that the anti-*Xylella* activity of strain C7 is largely dependent on the synthesis of VOCs. The mechanism of VOC-mediated antagonism is a well-documented phenomenon among plant-associated bacteria (Weisskopf et al., 2021), and this finding accentuates the potential significance of airborne bioactive molecules in the suppression of *Xfp*. The production of VOCs by C7 strain was further investigated by SPME and GC/MS. The analyses showed that 15 VOCs were produced in the headspace of the cultured strain C7 and are reported in Table 1. The identified VOCs belong to the chemical classes of esters, aldehydes, ketones, alcohols and short chain fatty acids as the results of microbial catabolism. Considering the area percentage, the most abundant compounds were ethyl acetate, butyl acetate, isoamyl acetate among esters and two short chain fatty acids, acetic and valeric acids. Even if SPME is generally considered not trustworthy for quantitative analysis, the area of each peak in the chromatogram is certainly correlated with the concentration of VOCs in the headspace. Therefore, by comparing the total area of the identified peaks in the headspace of the cultured strains during the incubation period, it is noteworthy that the production rate of VOCs in the investigated strain was very fast, reaching a constant level at 48 h of incubation (Figure 6).

**Table 1:** List of volatile organic compounds identified in the headspace of *D. fangzhongdai* strain C7 cultures after 24, 48 and 72 h of incubation.

RT	RI	Compound	Incubation time		
			24 h	48 h	72 h
1.98	831	Ethyl acetate	9.7b	16.6a	18.7a
2.20	881	Propyl acetate	0.6	0.7	0.7
2.34	910	2-methypropyl acetate	0.1	0.2	0.2
2.50	964	Butyl acetate	40.1a	29.5b	25b
2.77	1,015	Isoamyl acetate	14.7b	17.4a	16.3a
2.88	1,037	Hexanal	1.4	0.1	0.8
3.20	1,100	2-methyl butanol	8.8	8.8	7.3
3.44	1,144	3-methyl, 3-buten-1-ol	0.3	1.1	0.4
3.72	1,198	Butanone, 3-hydroxy	1.6b	1.5b	4.2a
4.79	1,446	Acetic acid	11.9b	15.5a	16.8a
5.31	1,547	Propionic acid	0.3	0.2	0.2
5.46	1,573	1,3-Butanediol	2.1	2.2	3.4
5.81	1,633	Butyric acid	0.9	0.7	0.8
6.06	1,674	Valeric acid	8.1a	5.2b	6.5ab

RT, retention time; RI, retention index. Values are the mean area percentages of relative peak obtained from the total ion chromatogram. Different letters in the same line represent statistical significance (*p* < 0.05) obtained by one way ANOVA.

### 3.4 Pathogenicity tests

**Figure 7:**
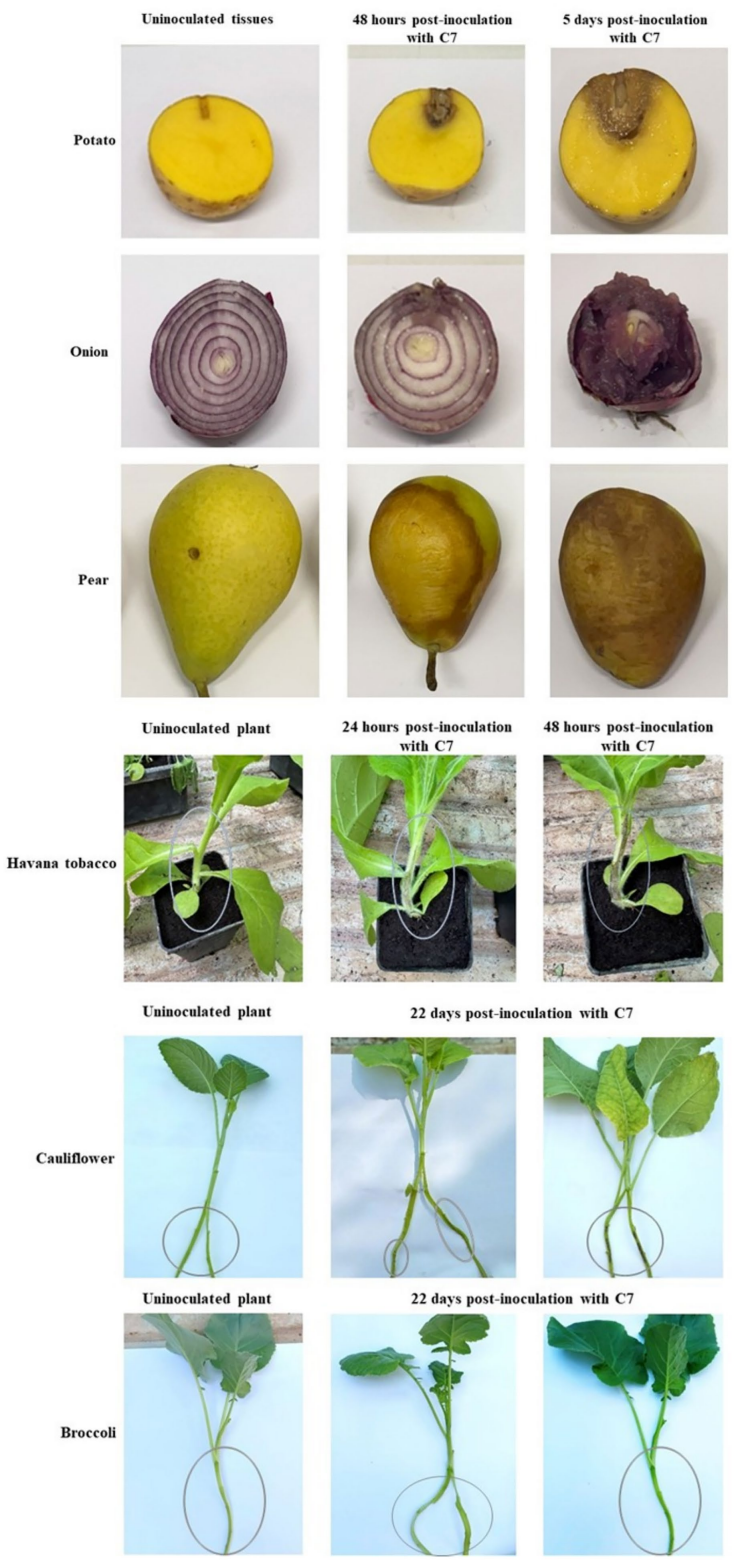
Pathogenicity assay showing the disease symptoms caused by *Dickeya fangzhongdai* strain C7 on various host plant tissues. Inoculated pear fruits, potato tubers, and onion bulbs exhibited characteristic soft rot symptoms, including tissue maceration and water-soaking. Tobacco plants developed blackleg symptoms, and cauliflower plants showed vascular tissue browning. In contrast, broccoli plants remained symptomless under the tested conditions.

**Figure 8:**
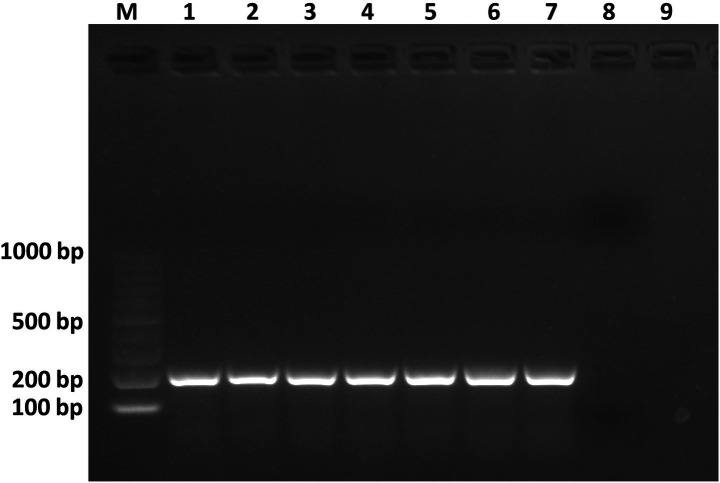
Agarose gel electrophoresis showing PCR products amplified from genomic DNA of *Dickeya fangzhongdai* strain C7 reisolated from cauliflower and broccoli plants 22 dpi. Lane M: 100 bp DNA ladder. Lanes 1–3: C7 reisolated from broccoli plants. Lanes 4–6: C7 reisolated from cauliflower plants. Lane 7: C7 used as positive control. Lane 8: sterile water used as a negative control reaction. Lane 9: healthy plant.

The pathogenicity assessment of strain C7 showed that all inoculated tissues exhibited rapid and progressive symptom development, except for broccoli plants, which remained asymptomatic (Figure 7). Tobacco stems showed initial blackleg and necrotic lesions within 24 h post-inoculation, evolving into extensive tissue damage by 48 h (Figure 7). Potato tubers, pear fruits, and onion bulbs demonstrated the onset of symptoms at approximately 48 h, characterized by localized softening and water-soaked lesions (Figure 7). By day 5 post-inoculation, all tissues exhibited severe maceration, accompanied by a strong, putrid odor, characteristic of advanced bacterial decomposition (Figure 7). Furthermore, C7-inoculated cauliflower plants developed symptoms characterized by vascular tissue browning by day 22 (Figure 7). Furthermore, strain C7 was successfully reisolated from inoculated cauliflower and broccoli plants on YPGA plates at 22 dpi (data not shown), and PCR assays of purified colonies confirmed the presence of the strain (Figure 8). These findings highlight the high virulence of strain C7 and its broad host tissue aggressiveness, underscoring its phytopathogenic potential across multiple economically relevant plant species.

## 4 Discussion

The escalating prevalence of *Dickeya* species across various agroecological environments constitutes a significant concern for global agricultural productivity. Our investigation presents the first report on the presence of *D. fangzhongdai* in Italy, that was isolated from cauliflower and subsequently demonstrated to affect five crops of considerable economic importance, i.e., Havana tobacco, potato, pear, cauliflower, and onion. These results corroborate the expanding host range and ecological adaptability of *D. fangzhongdai*, which has been previously documented on Asian pear, mango, orchid, banana, and taro in geographically disparate regions (Tian et al., 2016; Huang et al., 2021; Han et al., 2025). The virulence exhibited by strain C7 reflects a broader trend within the *Dickeya* genus, distinguished by its aggressive pathogenic capabilities enabled by a repertoire of plant cell wall-degrading enzymes (PCWDEs), including pectate lyases, cellulases, and proteases (Zhou et al., 2024; Babińska-Wensierska et al., 2025). Soft rot symptoms resulting from PCWDE activity not only leads to the degradation of plant tissue but also creates nutrient-enriched conditions that promote bacterial proliferation. When environmental conditions are favorable, particularly with elevated humidity and moderate temperatures, these mechanisms may result in swift epidemics that can precipitate yield losses surpassing 40% in specific crops (Elhalag et al., 2024).

The emergence of novel *Dickeya* species and strains such as *D. solani* and *D. fangzhongdai* that exhibit enhanced virulence and adaptability in temperate climates further escalates the likelihood of extensive outbreaks (Degefu et al., 2013; Curland et al., 2021). These bacteria can persist in irrigation water, soil, and asymptomatic planting material, and may be disseminated via insects and mechanical vectors (Charkowski, 2018). In our case, the capacity of *D. fangzhongdai* C7 to colonize and persist in both susceptible (cauliflower) and non-susceptible plant species (broccoli) was validated over a 22-day period. The persistence of *D. fangzhongdai* in diverse plant species, even asymptomatically, increases the likelihood of its undetected spread, enhances its dissemination potential, and accentuates the challenges for disease monitoring and containment. The molecular and bioinformatic analyses of the *D. fangzhongdai* C7 genome provided a comprehensive overview of the genomic landscape of this specific strain, which will help in the understanding of the molecular mechanisms of its pathogenicity, its adaptive characteristics, and the development of future diagnostic and control strategies for this emerging pathogen.

The antagonistic properties exhibited by strain C7 against *Xfp* observed in both direct-contact and physically separated culture experiments warrants particular attention, opening new perspectives for biological control of *Xfp*. The investigation about VOCs in the headspace of C7 strain pure culture highlighted the presence of compounds known for their antimicrobial activity. Ethyl and butyl acetate are proved to be effective in controlling the grow of *Pseudomonas aeruginosa, Staphylococcus aureus, Escherichia coli, Candida albicans and Trichophyton rubrum,* with minimum inhibitory concentration (MIC) < 5% (Lens et al., 2016). Ando et al. (2015) showed that the isoamyl acetate has a MIC below 0.2% against gram-positive, gram-negative bacteria and yeast. Acetic, propionic, butyric and valeric acids are characterized by a well know antibacterial activity and together with their derivates esters can act in deactivating the membrane potential of bacterial cells, thus impairing respiration. The *D. fangzhongdai* strain C7 seems to be able to produce a synergic mixture of VOCs, highly active in inhibiting the growth of *Xf*p. Compounds such as dimethyl disulfide, 2,3-butanediol, and acetoin, previously characterized in various antagonistic strains of phytopathogens, have been synthesized on industrial scale and utilized in agricultural practices (Russo et al., 2022). The synthesis of microbial VOCs can be simple and cost-effective, and for these reasons, if the identified active VOCs from strain C7 can be produced synthetically, they may serve as a new biopesticide useful in the integrated pest management of different diseases, among which *Xf* could be a target. Notwithstanding the encouraging results presented herein, several knowledge gaps need to be further addressed. First, the role of each identified VOC must be investigated, together with their antimicrobial synergisms, highlighting the most important and effective compounds. Then, an optimal mixture must be formulated, able to give the maximum antimicrobial activity at lowest concentration. Subsequently, *in planta* efficacy should be evaluated under greenhouse and open-field conditions to confirm bioactivity against *Xf* within a multifaceted ecological framework. Lastly, the biosynthetic regulation of VOC production, along with its modulation by environmental parameters, requires elucidation to enhance yield and stability under fluctuating conditions. Future research endeavors ought to take into consideration VOC-based strategies, including the utilization of resistant cultivars, management of vectors, and enhancement of diagnostic surveillance techniques.

In conclusion, this study reports for the first time the detection of *D. fangzhongdai* in Italy, demonstrating its ability to affect a wide range of economically important plant species. Notably, this pathogen was found to infect new hosts, i.e., cauliflower and Havana tobacco, further demonstrating its expanding host range and potential phytosanitary risk. This study also highlights the *D. fangzhongdai* properties, providing a theoretical basis for future research, effective prevention, and control of the disease.

## Data Availability

The datasets presented in this study can be found in online repositories. The names of the repository/repositories and accession number(s) can be found in the article/supplementary material.

## Footnotes

^1^ https://github.com/tseemann/prokka

^2^ https://crisprcas.i2bc.paris-saclay.fr/

^3^ https://phaster.ca/

^4^ https://www.pathogenomics.sfu.ca/islandviewer/browse/
